# Porous Se@SiO_2_ Nanoparticles Enhance Wound Healing by ROS-PI3K/Akt Pathway in Dermal Fibroblasts and Reduce Scar Formation

**DOI:** 10.3389/fbioe.2022.852482

**Published:** 2022-03-21

**Authors:** Bo-Yu Yang, Zhi-Yuan Zhou, Shi-Yun Liu, Ming-Jun Shi, Xi-Jian Liu, Tian-Ming Cheng, Guo-Ying Deng, Ye Tian, Jian Song, Xuan-Hao Li

**Affiliations:** ^1^ Department of Urology, Beijing Friendship Hospital, Capital Medical University, Beijing, China; ^2^ Shanghai Pudong New Area GongLi Hospital, Shanghai, China; ^3^ Department of Urology, Shanghai General Hospital, Shanghai Jiao Tong University School of Medicine, Shanghai, China; ^4^ College of Chemistry and Chemical Engineering, Shanghai University of Engineering Science, Shanghai, China; ^5^ Trauma Center, Shanghai General Hospital, Shanghai Jiaotong University School of Medicine, Shanghai, China

**Keywords:** porous se@SiO_2_ nanoparticles, wound healing, fibrosis, oxidative stress, PI3K/akt pathway

## Abstract

Hypertrophic scarring, which is characterized by excessive extracellular matrix deposition and abnormal fibroblast homeostasis, is an undesirable outcome of dermal wound healing. Once formed, the scar will replace the normal function of local skin, and there are few noninvasive clinical treatments that can cure it. Se@SiO_2_ nanoparticles were synthesized to suppress oxidative stress, which induced the presence and activation of myofibroblasts during wound recovery. The characterization, antioxidant capacity and biological safety of Se@SiO_2_ NPs were evaluated. A full-thickness excisional wound model was established, and the wounds were divided into three groups. The re-epithelization and distribution of collagen fibers were assessed using hematoxylin and eosin staining and Masson’s trichome staining after specific treatments. Our results revealed that the Se@SiO_2_ NPs accelerated dermal wound healing and suppressed the formation of hypertrophic scars, accompanied by oxidative stress inhibition. Moreover, we found that Se@SiO_2_ NPs worked by activating the PI3K/Akt pathway and upregulating the phosphorylation of Akt. The findings of our study provide a new method to promote dermal scar-free wound healing by suppressing excessive oxidative stress and through PI3K/Akt pathway activation.

## 1 Introduction

Skin is the largest immune organ in the human body and plays a vital role in resisting external pathogen stimulation (Chambers and Vukmanovic‐Stejic 2020). When traumatized or burned, the skin initiates a series of coordinated and orderly cellular biological responses to restore primary normal status, which is called wound healing. Wound healing is well described as a physiological process composed of three consecutive and overlapping stages, including the inflammatory response, granulation formation and tissue remodeling (Gurtner et al., 2008). There are many factors that can affect dermal wound repair, such as wound size, infection and immune function (Tatara et al., 2018). In terms of structure, the skin can be divided into three layers from outside to inside, namely, the *epidermis*, dermis and hypodermis. When skin damage reaches the dermis, fibrotic repair involves the participation of multiple cell types, such as fibroblasts, keratinocytes and macrophages, and excessive extracellular matrix (ECM) deposition occurs (Driskell et al., 2013; Ogawa 2017). The occurrence of excessive fibrosis is a key issue that affects the quality of skin wound repair (Finnerty et al., 2016). In addition, studies have shown that the downregulation of fibrosis-related genes can directly alleviate scar formation but delay wound healing (Chogan et al., 2020). Therefore, seeking strategies that act on these dual roles to solve the problem of fibrosis is the focus of promoting wound repair and an urgent need.

Fibrosis, which results in hypertrophic scarring, refers to the pathological process of parenchymal cell necrosis and excessive ECM deposition in tissues. It has been demonstrated that myofibroblasts have a strong ECM secretory function and are the activated form of resident wound tissue fibroblasts (Zhang et al., 2020). As the most important effector cell associated with scar formation, myofibroblasts are usually activated during the inflammatory response (Woodcock et al., 2019; Wu et al., 2019). Multiple factors in the wound microenvironment can initiate fibroblast differentiation into myofibroblasts (Wang et al., 2020). Reactive oxygen species (ROS) generation is another major event associated with tissue fibrosis (Park et al., 2015). ROS such as hydrogen peroxide (H_2_O_2_), which is known to be an essential part of the early stage of wound healing, play pivotal roles in fibroblast activation and fibrosis (Akasaka et al., 2021). Moreover, high levels of ROS, which induce oxidative stress *in situ*, could hinder wound healing (Cheng et al., 2021). There is also evidence that low levels of ROS have positive effects on cell proliferation rather than promoting apoptosis (Dunnill et al., 2017; Schmidt et al., 2021). Therefore, topical administration of a drug with a sustained-release effect to maintain ROS at beneficial levels is particularly needed.

As one of the main ingredients in common antioxidant drugs, selenium (Se) is an essential trace element in the human body and a powerful antioxidant that works through glutathione peroxidase (GSH-Px) to scavenge free radicals and repair cell damage (Schrauzer 2009; Hariharan and Dharmaraj 2020). Se is seldom applied in the clinic because of its narrow therapeutic window (Clark et al., 1996; Alkie et al., 2020). In recent years, due to the development of nanomedical technology and drug delivery systems, Se particles have been modified for several generations, and finally porous Se@SiO_2_ nanoparticles (Se@SiO_2_ NPs) with excellent slow release capacity and biosafety were synthesized (Liu et al., 2016; Wang et al., 2018; Yang et al., 2019). Our previous study showed that porous Se@SiO_2_ nanosphere-coated catheters could promote prostatic urethral wound healing (Yang et al., 2019). Thus, we hypothesized that Se@SiO_2_ NPs could both promote dermal wound recovery and reduce scarring. In our study, we successfully synthesized Se@SiO_2_ NPs and dissolved them in a chitosan (CS)/acetic acid solution. A full-thickness excision wound model on the backs of rats was established. The properties and biosafety of this nanocomposite were characterized, and the re-epithelization of wound tissues and degree of fibrosis were investigated. In addition, we explored the specific molecular mechanism by which Se@SiO_2_ NPs promote scar-free wound repair. The results of this study suggested that the application of Se@SiO_2_ NPs effectively enhanced dermal scar-free wound healing.

## 2 Materials and Methods

### 2.1 Reagents and Antibodies

H_2_O_2_ (30%) was obtained from Sigma (St Louis, MO, United States). Antibodies targeting α-SMA (ab5694), fibronectin (ab2413) and Col1A (ab96723) were purchased from Abcam (Cambridge, United Kingdom). Akt (sc-8312) and PARP/c-PARP (sc-7150) antibodies were purchased from Santa Cruz Biotechnology (Santa Cruz, CA, United States). GAPDH (#2118), phospho-Akt (#4060), and caspase-3/c-caspase-3 (#9665T) antibodies, horseradish peroxidase (HRP)-linked anti-rabbit IgG (#7074) and HRP-linked anti-mouse IgG (#7076) were purchased from Cell Signaling Technology (Danvers, MA, United States).

### 2.2 Synthesis of Se@SiO_2_ Nanoparticles

**SCHEME 1:**
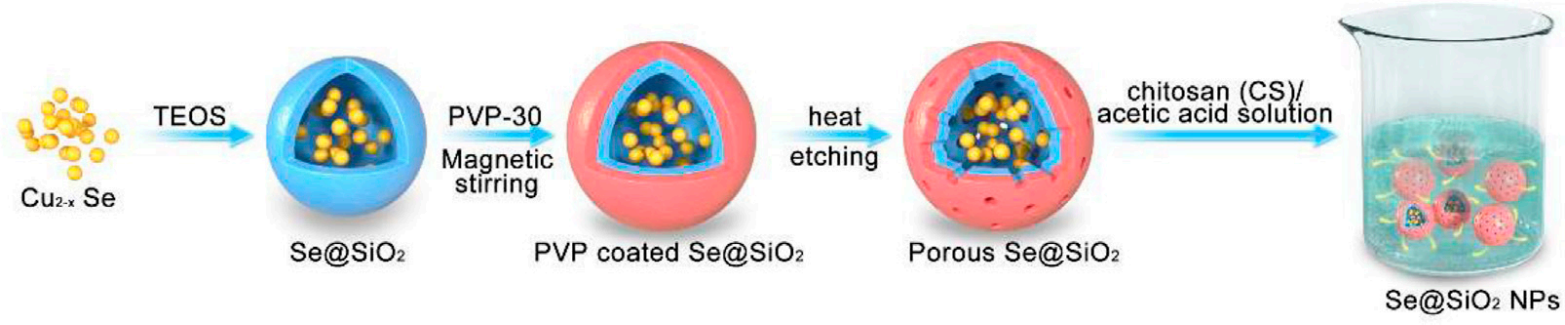
Schematic diagram of Se@SiO_2_ NPs synthesis.

Porous Se@SiO_2_ nanospheres were synthesized according to a previously reported process (Liu et al., 2016; Wang et al., 2018). Cu2-xSe nanocrystals were first prepared by the thermal injection method. Then, n-hexanol, Triton X-100, n-hexane, deionized water, TEOS and ammonia were added individually to the solution dropwise while stirring, according to the one-pot method. After being kept at room temperature for 24 h, Se@SiO_2_ core-shell nanospheres were collected by the addition of ethanol and were dispersed in deionized water. PVP-K30 was added and incubated for 1 h with magnetic stirring. The reaction mixture was heated to 95°C for 2 h and then rapidly cooled to 60°C. Porous Se@SiO_2_ nanospheres were collected by centrifugation and washed twice with ethanol. First, 0.1 g of CS was dissolved in 20 ml of 1% acetic acid solution with stirring. Then, the porous Se@SiO_2_ nanospheres were mixed with the solution after being dried, and we finally obtained Se@SiO_2_ NPs (Scheme 1). The mixture was evenly stirred and sealed for later use.

### 2.3 X-Ray Diffraction and Transmission Electron Microscopy

X-ray diffraction (XRD) analysis of the Se and m Se@SiO_2_ NPs was performed using an X-ray diffractometer (D/max-2550, Rigaku, Japan) in the range of 10°–80° (2θ) to detect phase consistency. The mean diameter, structure and morphology of the Se@SiO_2_ NPs were examined by transmission electron microscopy (TEM, JEM-2100F, JOEL, Japan). ImageJ was used to determine the size of the dried Se@SiO_2_ NPs.

### 2.4 Establishment of a Dermal Full-Thickness Excisional Wound Model

Twelve 1-year-old Sprague-Dawley rats weighing 250–300 g were obtained from Shanghai General Hospital. After a few days of adaptation to the laboratory environment, the backs of the rats were shaved to expose the area to be wounded and cleaned with phosphate-buffered saline (PBS). To establish a dermal full-thickness excisional wound model, we anaesthetized the animals with pentobarbital sodium and utilized a 1 cm sterile drill to remove skin tissues to the depth that adipose tissues were exposed. The four wounds were divided into three experimental groups: a “(-) group” (the wound in the top left corner was treated with PBS), a “SiO_2_ group” (the wound in the bottom left corner was treated with SiO_2_ nanospheres only), and a “Se@SiO_2_ group” (the two wounds on the right were treated with 1 mg/ml Se@SiO_2_ NPs). The wounds were covered with gauze bandages after the specific treatments, and each rat was tagged with ear studs. The rats were housed in groups and allowed food and water normally after the surgery. The rats were sacrificed at 1, 3, 7, 14, 21, and 28 days. The wound tissues were excised using surgical scissors and dissected into two equal parts. Venous blood (10 ml) was collected and centrifuged at 3,000 rpm for 20 min. The serum was stored at −80°C for further use. In addition, control groups were established by treatment of PBS to all four wounds on the back of rats. Animal experiments were authorized by the Medical Science Ethics Committee of Shanghai General Hospital and performed according to the principles of the Chinese Council on Animal Care. All procedures were in accordance with the ethical standards of The Medical Ethics Committee of Beijing Friendship Hospital Affiliated to Capital Medical University. All institutional and national guidelines and rules for the care and use of laboratory animals were followed.

### 2.5 Flow Cytometry

Cell apoptosis was examined using a FITC-Annexin V Apoptosis Detection Kit (BD Biosciences, CA, United States). Flow cytometry was used to assess the proportion of apoptotic cells after the different treatments according to the manufacturer’s instructions. Briefly, after being cultured with 300 μM H_2_O_2_ or 30 μg/ml Se@SiO_2_ NPs for 24 h, the cells were harvested with scrapers. After being washed twice with cold PBS (HyClone), the cells were resuspended in binding buffer and stained with Annexin V-FITC and PI solutions in the dark. Then, the cell suspensions were analyzed using a BD Accuri C6 flow cytometer (BD Biosciences, CA, United States).

### 2.6 Quantitative Real-Time Polymerase Chain Reaction

Total RNA was extracted from human skin fibroblasts (HSFs) using TRIzol reagent (Invitrogen, United States). Complementary DNA was synthesized using SuperScript III Reverse Transcriptase (Invitrogen, United States) according to the manufacturer’s guidelines. qRT-PCR was conducted using PowerUp™ SYBR^®^ Green Master Mix (Thermo Scientific, Waltham, MA, United States). Relative mRNA expression was quantified using the 2−ΔΔCt method and normalized to GAPDH as an internal control. The primers used in this study are listed in Supplementary Table S1.

### 2.7 Western Blotting

Cells were lysed with RIPA buffer (Beyotime, Suzhou, China) containing a phosphatase inhibitor cocktail (Yeasen, Shanghai, China) on ice for 30 min after the treatments and then vibrated by ultrasound. Protein levels were quantified using a Pierce BCA protein assay kit (Thermo Scientific, Waltham, MA, United States). Duplicate quantities of protein were loaded and separated on 10–15% SDS-polyacrylamide gels. Then, the proteins were transferred onto polyvinylidene fluoride (PVDF) membranes (Millipore, Bedford, MA, United States). After being blocked with skimmed milk for 1.5 h at room temperature, the membranes were incubated with specific primary antibodies overnight and HRP-conjugated secondary antibodies for 1.5 h after being washed with Tris-buffered saline plus Tween-20 (TBST). The immunoreactive bands were visualized using ChemiLucent ECL reagent (Millipore) and ImageJ software (National Institutes of Health).

### 2.8 Cell Viability Assay

The viability of HSFs was detected using Cell Counting Kit-8 (CCK-8, Dojindo, Japan). Cells were plated in 96-well plates at a density of 1 × 10^4^/well. After being treated with different concentrations of Se@SiO_2_ NPs for 24 h, HSFs were incubated with 10 μl of CCK-8 reagent for an additional 2 h. Then, the absorption at 450 nm was measured using a microplate reader.

### 2.9 Liver and Kidney Function Analysis

Serum was collected from rats at each time point to analyze liver and kidney functions, including ALB, ALP, ALT, AST, BUN, Cre, γ-GT, UA and TBIL, using a Chemray 240 automatic biochemical analyzer (Rayto Life and Analytical Sciences, Shenzhen, China).

### 2.10 Histology and Immunofluorescence

The wound tissues were fixed in 10% formalin for 12 h, embedded in paraffin and cut into 5 μm-thick sections. Hematoxylin and eosin (H&E) staining and Masson’s trichrome staining were performed. To determine the expression of α-SMA, immunofluorescence assays were also performed. The sections were treated with blocking buffer and then incubated with primary antibodies targeting α-SMA overnight at 4°C and secondary antibodies with fluorescent conjugates for 1.5 h at room temperature. Finally, the sections were washed with PBS plus Tween-20 (PBST) three times and incubated with DAPI for nuclear staining before being observed using a Leica DMi8 fluorescence microscope or a Leica TCS SP8 confocal microscope (Wetzlar, Germany).

### 2.11 DCFH-DA Assay

HSFs were seeded in a 35 mm confocal dish (Coverglass Bottom Dish; 5 × 105 cells/well) and pretreated with 30 μg/ml Se@SiO2 NPs for 24 h, followed by stimulation with 300 μM H_2_O_2_ or ddH_2_O for 30 min. Next, the HSFs were washed three times with cold PBS and incubated with DCFH-DA (Beyotime) for 15 min at 37°C. The dishes were screened using a Leica TCS SP8 confocal microscope (Wetzlar, Germany).

### 2.12 Wound Healing Assay

HSFs were seeded evenly in 6-well plates. We made an artificial wound in the cells using a plastic pipette tip when the HSFs had formed a monolayer. Then, the HSFs were cultured in the same culture medium and specific treatments until they reached 90–100% confluence relative to the primary wound. Cell migration at each time point was recorded by photography.

### 2.13 EdU Assay

Cell proliferation was analyzed using a Cell-LightTM EdU DNA Cell Proliferation Kit (RiboBio, Guangzhou, China) according to the manufacturer’s instructions. The results were observed using a fluorescence microscope, and the ratio of positive cells was calculated.

### 2.14 Statistical Analysis

Statistical analysis was performed using SPSS 19.0.0 software (SPSS Inc., Chicago, IL, United States). All data are expressed as the means ± standard deviation (SD) and were analyzed using a two-tailed Student’s *t*-test or one-way ANOVA. A *p*-value <0.05 was considered statistically significant. All experiments were performed in triplicate.

## 3 Results

### 3.1 Production, Particle Size and Patterns of Se@SiO_2_ Nanoparticles

**FIGURE 1:**
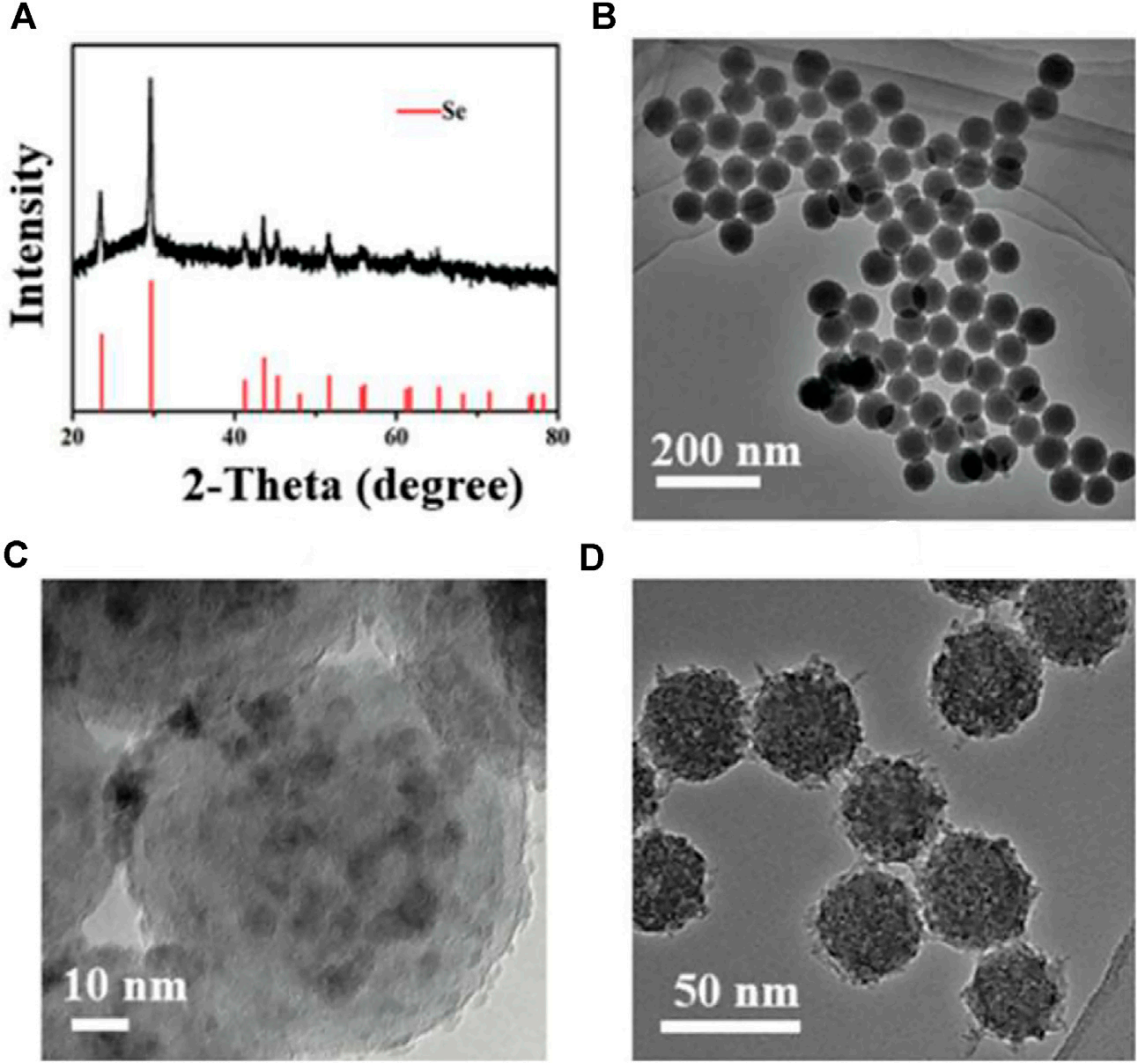
Characterization of the Se@SiO_2_ NPs. **(A)** The XRD spectra of the Se@SiO_2_ NPs and the standard Se hexagonal phase (JCPDS card number: 65–1876). **(B)** Low- and **(C)** high-magnification images of Se@SiO_2_ nanoparticles. **(D)** TEM characterization of the synthetic Se@SiO_2_ NPs. TEM microphotographs indicated the structure of Se@SiO_2_ NPs which consist of a porous silicon shell and encapsulated Se. Abbreviations: Se@SiO_2_ NPs, porous Se@SiO_2_ nanoparticles; XRD, X-ray diffraction; TEM, transmission electron microscopy; Se, selenium.

The XRD pattern of Se@SiO_2_ NPs is shown in Figure 1A. The characteristic (1 0 0), (0 1 1), (1 1 0) and (0 1 2) peaks demonstrated that the Se@SiO_2_ NPs matched the standard hexagonal phase of Se (JCPDS card number 65–1876). The XRD analysis indicated that the presence of the amorphous silica coating showed a steady increase in the baseline of the low-angle region. The average diameter of the Se@SiO_2_ nanoparticles as detected by TEM was approximately 55 nm. The Se@SiO_2_ nanoparticles were monodispersed in a uniform spherical structure with Se quantum dots distributed from the center of the nanosphere to the surface (Figures 1B,C). After they were immersed in the PVP solution and heat-treated to form a porous silicon shell, Se@SiO_2_ NPs were synthesized (Figure 1D). Finally, Se@SiO_2_ NPs were dissolved in the carrier CS solution for practical use to ensure sustained release.

### 3.2 Promotion of Excisional Wound Closure With Wound Contracture and Dermal Morphology Optimization in Rats

**FIGURE 2:**
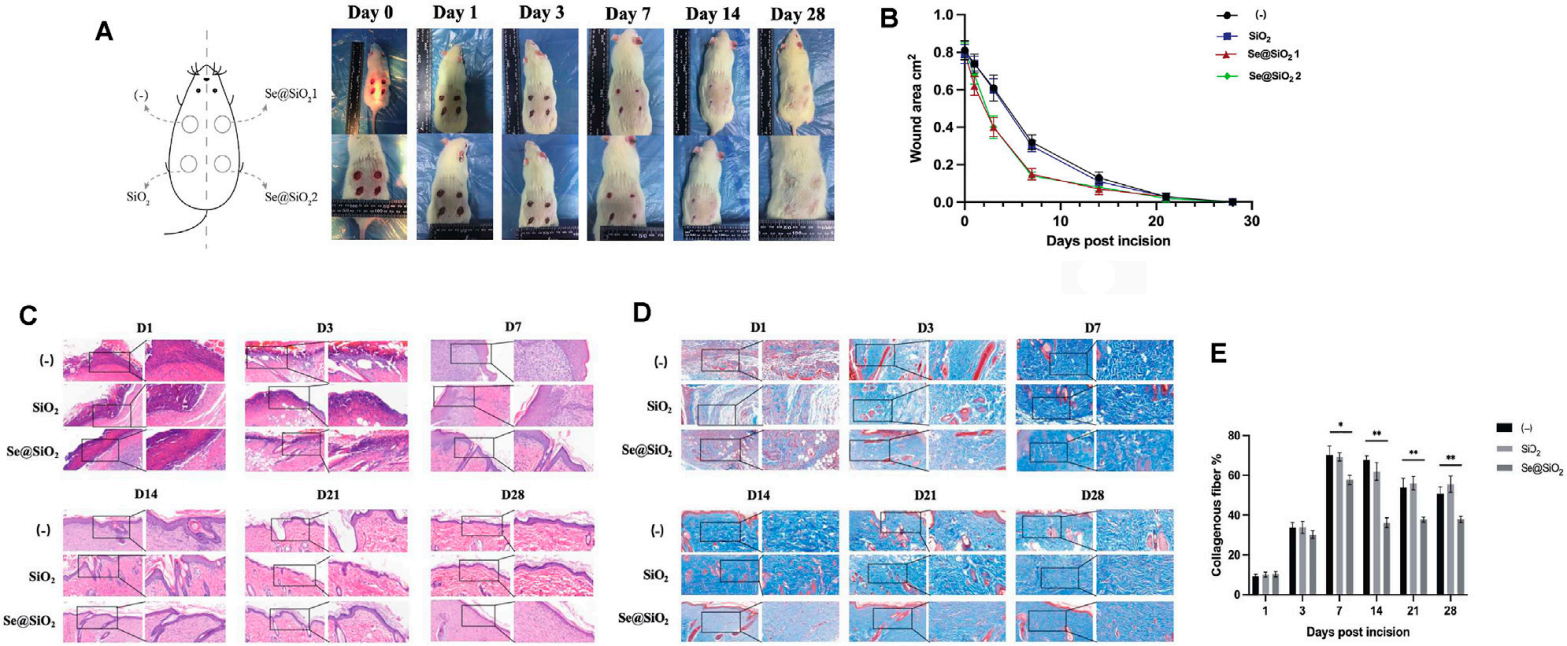
Se@SiO_2_ NPs promote the process of scarless wound healing. **(A)** Establishment of full-thickness wounds on the back of rats. **(B)** Wound closure rate of excisional injury. **(C)** H&E staining shows changes of dermal wound surface reconstruction in the Se@SiO_2_, SiO_2_ and (-) groups at each time point (scale bars: 50 μm) (200× and 400×). **(D)** Masson’s trichrome staining of wound tissues at each time point. **(E)** Density of collagen in the wound tissues based on Masson’s trichrome staining. ^*^ *p* < 0.05, ^**^ *p* < 0.01 and ^***^ *p* < 0.001. Abbreviations: H&E, hematoxylin and eosin.

Since the dermal full-thickness wound model was established with a drill, excisional wounds on the backs of the rats were divided into three groups. The progression of wound repair was recorded according to the scheduled observation times (Figure 2A). During the first 3 days, there was no difference in the groups. On day 3, the Se@SiO_2_ group exhibited an accelerated repair process in terms of the wound area, and the wounds in the (-) group and SiO_2_ group were moist with fresh granulation tissue. On day 7, the wound healing speed of Se@SiO_2_ group achieved almost 80%, which was pivotally faster than that of (-) group and SiO_2_ group (*p* < 0.01). Eventually, the wound in the Se@SiO_2_ group healed thoroughly by day 21. The Se@SiO_2_ NPs promoted flat closure that was fully filled in the cavity and almost the same as the adjacent normal skin. In contrast, wounds in the (-) group closed on day 28, but the palpable hypertrophic scar was reddish and obviously raised, which was similar to that in the SiO_2_ nanosphere-treated group. Measurement of the wound area confirmed that Se@SiO_2_ NPs could accelerate wound healing (Figure 2B). To demonstrate the effect of Se@SiO_2_ NPs histologically, we performed H&E staining at each time point (Figure 2C). One week after the dermal full-thickness wound model was established, we observed a more actively proliferating epithelium consisting of one or two layers of cells in both Se@SiO_2_ groups. By week 4, histological analysis indicated that both Se@SiO_2_ groups developed a continuous regenerative epithelium; moreover, well-formed basal lamina layers arranged in distinct levels were observed compared to those in the (-) group and SiO_2_ group. Masson’s trichrome staining was used to assess the degree of wound fibrosis (Figure 2D). Consistently, we found that the density of collagen deposition in both Se@SiO_2_ groups was markedly reduced compared to that in the (-) and SiO_2_ groups at the fourth week (Figure 2E). Thus, our results confirmed that Se@SiO_2_ NPs promoted dermal wound healing with accelerated wound closure, optimized morphology and reduced hypertrophic scar formation.

### 3.3 Evaluation of Se@SiO_2_ Nanoparticles Release Profiles, Biosafety and Oxidation Resistance

**FIGURE 3:**
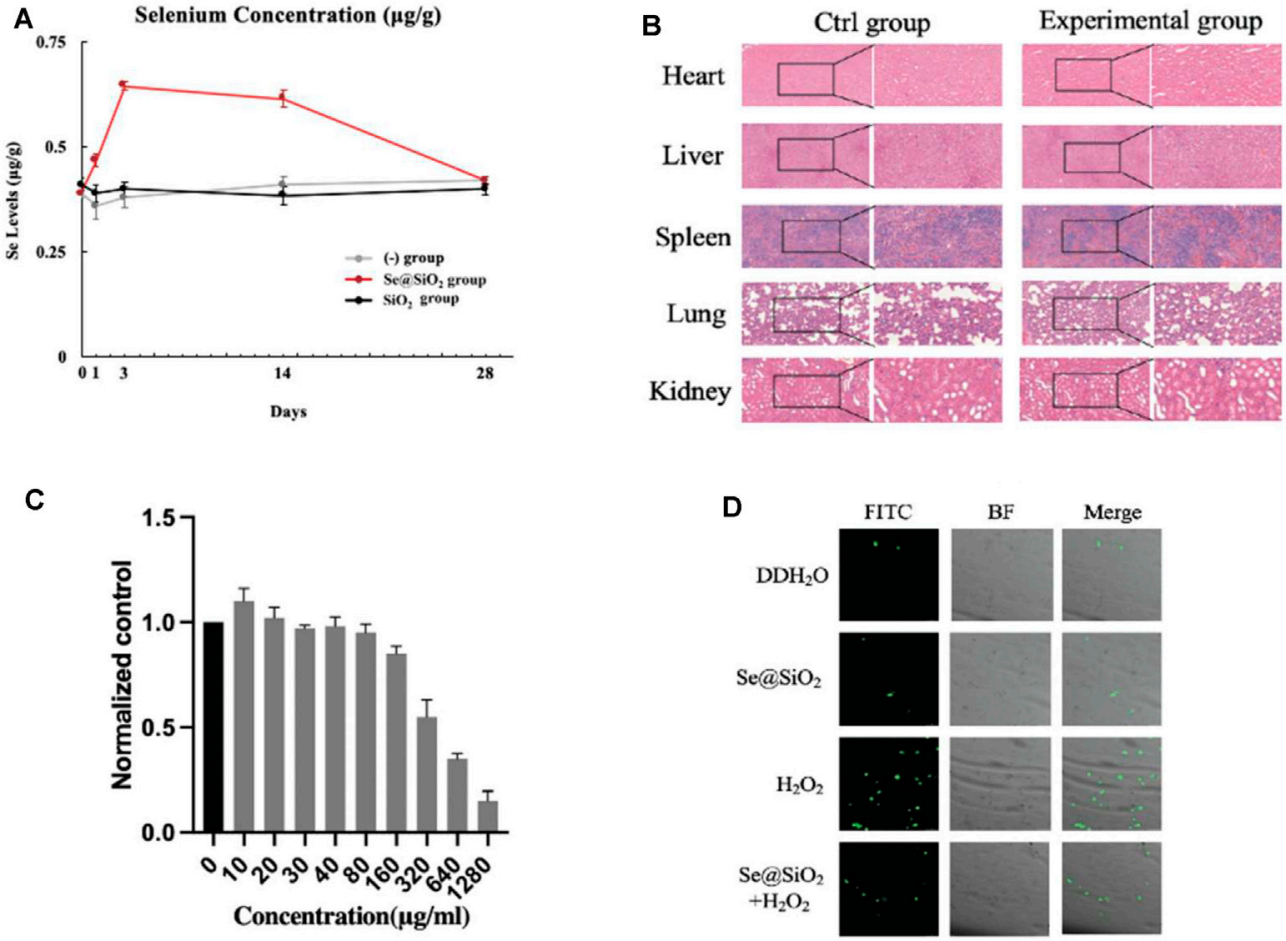
Release profile, biosafety and oxidation resistance of Se@SiO_2_ NPs *in vivo* and vitro. **(A)** ICP-AES defined the Se contents in regenerated tissues of dermal wound at each time point. **(B)** Histological analysis of the heart, liver, spleen, lung and kidney tissues of rats at 4 weeks after the *in vivo* models were established (scale bars: 50 μm) (200× and 400×). **(C)** The viability of HSFs was detected after 24 h of coculture with different concentrations of porous Se@SiO_2_ nanoparticles using a CCK-8 assay. **(D)** ROS expression was demonstrated by FITC images, BF images and merged views combining BF and FITC images using a DCFH-DA assay observed by confocal microscopy. ^*^ *p* < 0.05, ^**^ *p* < 0.01 and ^***^ *p* < 0.001. Abbreviations: ICP-AES, inductively coupled plasma atomic emission spectroscopy; HSFs, human scar fibroblasts cell line; CCK-8, Cell Counting Kit-8; ROS, reactive oxygen species; FITC, fluorescein isothiocyanate; BF, bright field; DCFH-DA, 2′,7′-dichlorodihydrofluorescein diacetate.

To describe the release spectrum of Se@SiO_2_ NPs, ICP-AES was used to evaluate the Se content in the wound area in the groups. The Se concentration in wound tissue in the Se@SiO_2_ group reached a maximum in the first week and remained steady thereafter (Figure 3A). As expected, there was no increase in Se in the (-) group or SiO_2_ group. Moreover, no significant increase in Se was detected in serum (Supplementary Table S1). Furthermore, abnormal behaviors in the rats, such as spasm, coma, vomiting, and incontinence, did not occur after full-thickness wound models were established, indicating the high biosafety of Se@SiO_2_ NPs. H&E staining of major organs was performed, and there was no histological difference among the groups (Figure 3B). Plasma samples were collected at each time point to examine liver and kidney function. As shown in Supplementary Table S2, the serum levels of ALB, ALP, ALT, AST, BUN, Cre, γ-GT, UA and TBIL in the experimental groups were not significantly changed compared to those in the control groups throughout the experiment; the experimental groups did not exhibit metabolic dysfunction. Before subsequent examinations, we also determined the safety of the Se@SiO_2_ NPs *in vitro*. The CCK-8 assay results demonstrated that concentrations ranging from 0 to 160 μg/ml were not significantly toxic to HSFs (Figure 3C). We simulated the oxidative stress environment in wound tissue by incubating HSFs with H_2_O_2_. HSFs were pretreated with Se@SiO_2_ NPs and SiO_2_ nanospheres, and then, a DCFH-DA assay and confocal microscopy were performed to investigate the ROS levels. Pretreatment with Se@SiO_2_ NPs effectively downregulated H_2_O_2_-induced ROS levels (Figure 3D). Therefore, the Se@SiO_2_ NPs decreased oxidative stress, which is crucial during wound healing, at safe concentrations and with a slow release.

### 3.4 Se@SiO_2_ Nanoparticles Suppress Oxidative Stress-Induced Dermal Fibroblast Activation

**FIGURE 4:**
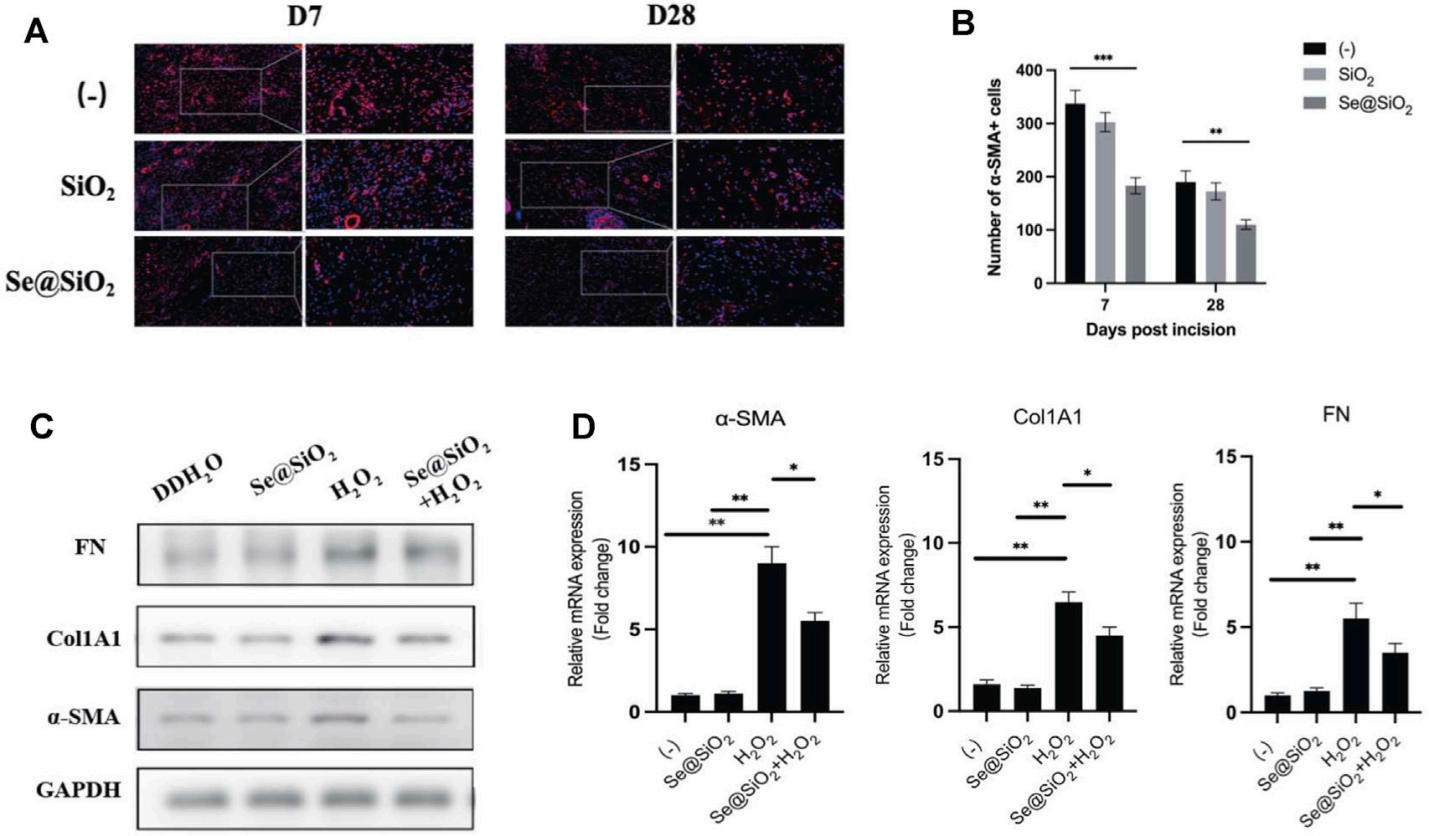
Se@SiO_2_ NPs repress collagen deposition of dermal fibroblasts caused by oxidative stress. **(A)** Representative images of immunofluorescence for α-SMA (red) in wound tissues at Day 7 and Day 28 (scale bars: 50 μm) (200× and 400×). Nuclei were stained with DAPI (blue). **(B)** Statistical analysis of α-SMA + cells in immunofluorescence images. **(C)** Western blot analysis of relative α-SMA, Col1A1 and FN expression levels with Se@SiO_2_ NPs and H_2_O_2_ treatment. **(D)** The mRNA levels of the α-SMA, Col1A1 and FN were further detected using qRT-PCR (*n* = 3). ^*^ *p* < 0.05, ^**^ *p* < 0.01 and ^***^ *p* < 0.001. Abbreviations: α-SMA, alpha smooth muscle actin; DAPI, 4′,6-diamidino-2-phenylindole; Col1A1, collagen 1A1; FN, fibronectin; qRT-PCR, quantitative real-time polymerase chain reaction.

Considering that better outcomes such as scarless wound healing were observed histologically in the Se@SiO_2_ group, we further explored the activation of fibroblasts in this process. Immunofluorescence staining showed that in wound tissues, α-SMA, a biomarker of activated fibroblasts, was highly expressed in the (-) group and SiO_2_ group but was decreased in the Se@SiO_2_ group (Figure 4A, B). Incubation with H_2_O_2_ markedly upregulated α-SMA protein expression in HSFs. The expression of type I collagen and fibronectin was also examined to evaluate whether Se@SiO_2_ NPs modulated the accumulation of ECM, which was highly enhanced by fibroblast activation (Figure 4C). The results confirmed that Se@SiO_2_ NPs could suppress the upregulated expression of α-SMA, fibronectin and Col1A1 proteins caused by H_2_O_2_, which are essential for scar formation. In addition, Se@SiO_2_ NPs also downregulated the mRNA expression of α-SMA, fibronectin and Col1A1 (Figure 4D). Our results showed that Se@SiO_2_ NPs reversed the profibrotic effect induced by oxidative stress *in vivo* and *in vitro*.

### 3.5 Se@SiO_2_ Nanoparticles Efficiently Attenuate ROS-Mediated Apoptosis via PI3K/Akt Activation

**FIGURE 5:**
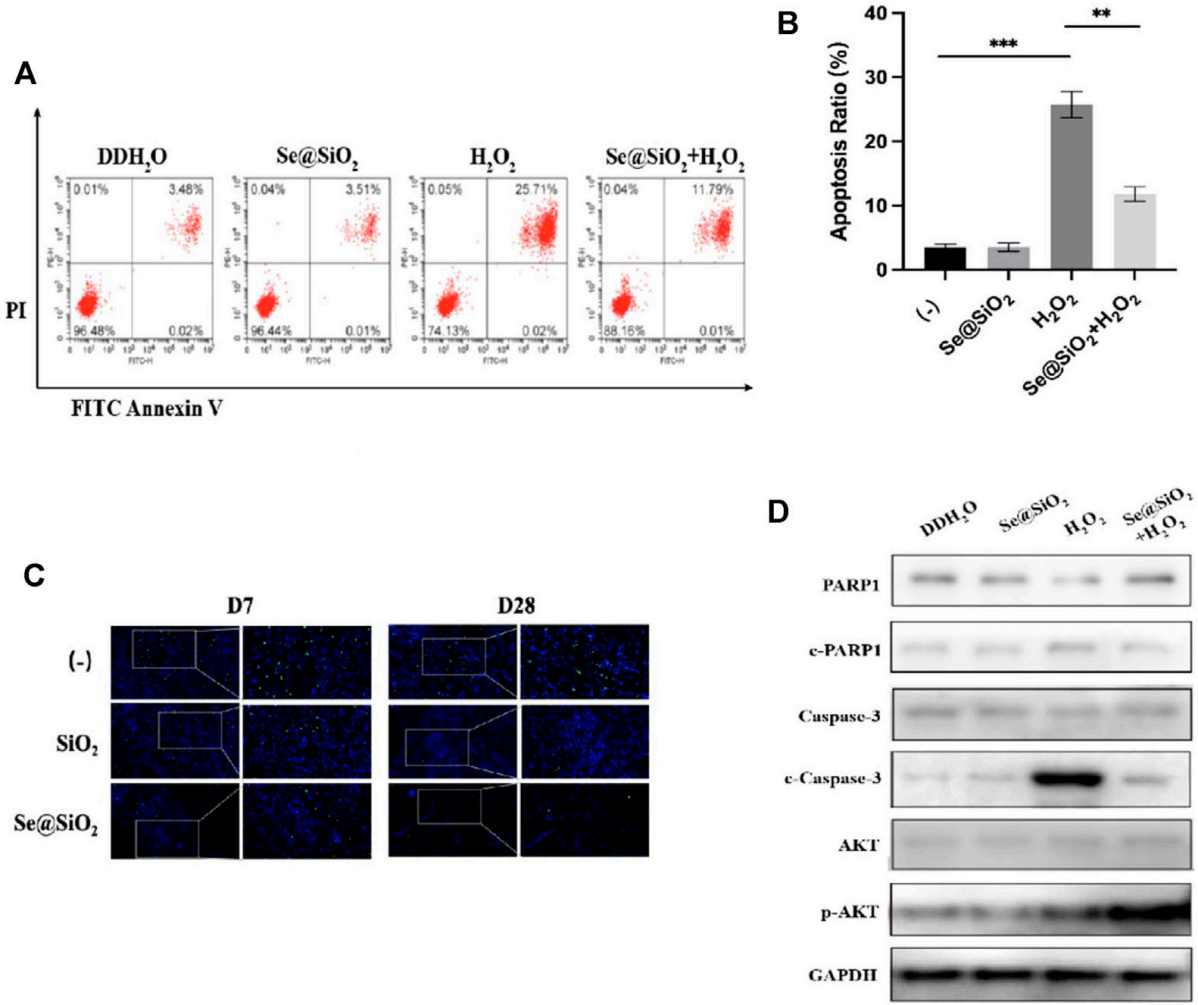
Se@SiO_2_ NPs can efficiently attenuate ROS-mediated apoptosis via PI3K/Akt activation. **(A)** Flow cytometry was conducted to detect the HSF apoptosis after pretreatment with 30 μg/ml Se@SiO_2_ NPs for 24 h, followed by coculture with 300 μM H_2_O_2_ or DDH_2_O for another 24 h. **(B)** Statistical analysis of the apoptotic cell percentage. **(C)** Immunofluorescence for TUNEL staining (green) was performed to validate the results of flow cytometry analysis. Nuclei were stained with DAPI (blue) **(D)** Relative expression levels of PARP1, c-PARP1, Caspase-3, c-Caspase-3, AKT and p-AKT proteins were detected via western blotting. ^*^ *p* < 0.05, ^**^ *p* < 0.01 and ^***^ *p* < 0.001. Abbreviations: PI3K/Akt, Phosphatidylinositol 3-kinase/Akt; DDH_2_O, double distilled H_2_O; TUNEL, TdT mediated dUTP Nick End Labeling; PARP1, poly ADP-ribose polymerase 1; c-PARP1, cleaved poly ADP-ribose polymerase 1; c-Caspase-3, cleaved Caspase-3; p-AKT, Phosphorylated Akt.

Next, we examined the mechanism by which Se@SiO_2_ NPs promoted wound healing. Flow cytometry showed that Se@SiO_2_ NPs abrogated the effect of H_2_O_2_ on apoptosis (Figures 5A, B). Additionally, TUNEL staining was used, and the results showed that the number of a-TUNEL + cells decreased in the Se@SiO_2_ group, which indicated that Se@SiO_2_ NPs suppressed oxidative stress-induced apoptosis in fibroblasts (Figure 5C).

PI3K/Akt has been reported to be a vital factor that regulates apoptosis, and the antiapoptotic effect of Se@SiO_2_ NPs on dermal fibroblasts has been verified. Thus, we examined whether Se@SiO_2_ NPs could regulate ROS-induced apoptosis by triggering the phosphorylation of Akt. We used Western blotting to measure the expression of total and phosphorylated Akt. As shown in Figure 5D, the expression of cleaved Caspase-3 and cleaved PARP1 decreased, and phospho-Akt was upregulated in the Se@SiO_2_+H_2_O_2_ group compared to the H_2_O_2_ group. In conclusion, Se@SiO_2_ NPs protected against ROS-mediated apoptosis in HSFs via PI3K/Akt activation.

## 4 Discussion

Wound healing is a continuous process with multiple stages, including the inflammatory response, granulation formation and tissue remodeling (Gurtner et al., 2008). As the most important part of initial wound formation, the inflammatory response, especially when accompanied by excessive oxidative stress, plays a vital role in wound healing. Our previous study revealed that the polarization of M1 macrophages to M2 macrophages by modulating ROS levels could shorten the proinflammatory stage and accelerate re-epithelialization (Yang et al., 2019). Fibroblasts, one of the main effector cells that facilitate repair, are generally activated during the inflammatory response. Studies have shown that oxidative stress is one of the initiators of fibroblast activation (Akasaka et al., 2021). The number of myofibroblasts is in dynamic balance. The major characteristic of dermal scarring is persistent myofibroblast activation, as well as ECM deposition (Hinz and Lagares 2020). Thus, preventing apoptosis and the differentiation of fibroblasts is a feasible strategy that can not only accelerate wound healing but also reduce scar formation.

In the present study, our team successfully synthesized Se@SiO_2_ NPs to promote scar-free dermal wound healing using a thermal injection method and a one-pot method as previously reported (Liu et al., 2016; Wang et al., 2018). Se, which is located in the core of Se@SiO_2_ NPs, has been recognized as a therapeutic agent against oxidative stress. Several studies have confirmed Se-mediated promotion of cell growth, but Se is difficult to apply clinically due to its narrow treatment concentration range. In the present study, we dissolved Se@SiO_2_ NPs in a solution of CS with good adhesion properties. The solution could prevent the NPs from easily falling off of the wound and prolong the action time. Then, we established a full-thickness incision model on the backs of rats by and coated the solutions directly on the wounds. Compared to systemic administration, directly coating the wound with Se@SiO_2_ NPs was faster and is safer since it allowed more flexible dosing control. Additionally, topical application of Se@SiO_2_ NPs does not enter the blood circulation, so damage to other major organs was also minimized.

Until now, there has been no definitively effective therapy to promote scar-free wound healing because of its unclear mechanism. Dermal fibroblasts in the early stage after skin injury are responsible for wound closure to some extent. Excessive and uncontrolled oxidative stress could lead to fibroblast apoptosis and delayed healing. As we expected, Se@SiO_2_ NPs effectively elevated the levels of endogenous antioxidants in wound tissues. Se@SiO_2_ NPs protected HSFs from ROS-induced apoptosis, and TUNEL staining confirmed this effect histologically. Caspase-3, an executioner caspase that functions through the heteroactivation pathway, plays an indispensable role in apoptosis; however, it has no catalytic activity itself. Once cleaved and activated, caspase-3 hydrolyses the target protein and initiates apoptosis (Esteban-Fernández de Ávila et al., 2017). PARP is considered to be a sensor of DNA damage and a primary cleavage target of Caspase-3. Cleaved PARP is an important indicator of cell apoptosis, and it is also generally regarded as an indicator of Caspase-3 activation (Chang et al., 2011; Bergamaschi et al., 2019). In the current study, we found that the expression of c-caspase-3 and c-PARP was upregulated by H_2_O_2_ and subsequently downregulated after Se@SiO_2_ NP administration.

The fibrosis degree in the recovered tissue is another important index to evaluate wound repair. Serious scarring can damage the normal structure and function of various tissues (Mor et al., 2019). The level of collagen fibers in scar tissue was significantly higher than that in adjacent normal skin tissue. In this study, the tissues treated with Se@SiO_2_ NPs exhibited less positive collagen fiber arranged in the dermis than the (-) group, as shown by Masson’s staining. As collagen and fibronectin are key components of the ECM, the overexpression of Col1 and fibronectin is one of the typical characteristics of ECM deposition that is distinct from normalized scar-free tissues (Patten and Wang 2021; Russo et al., 2021; Witherel et al., 2021). Furthermore, we explored the expression of myofibroblast cell marker α-SMA in fibroblasts treated with Se@SiO_2_ NPs. The elevated α-SMA expression in fibroblasts induced by H_2_O_2_ was abolished after the pretreatment of Se@SiO_2_ NPs. Therefore, our results demonstrated Se@SiO_2_ NPs didn’t stimulate the trans-differentiation of fibroblasts into myofibroblasts.

The mechanism by which the inhibition of excessive ROS by Se@SiO_2_ NPs reduced HSF apoptosis and differentiation remains unclear. Studies have shown that the PI3K/Akt pathway plays a pivotal role in cell survival (Franke et al., 1997). We found that treatment with Se@SiO_2_ NPs increased the expression of p-Akt, which confirmed the activation of the PI3K/Akt pathway. Combined with the ROS scavenging capacity of Se@SiO_2_ NPs, our study indicated that Se@SiO_2_ NPs could reverse ROS-induced apoptosis through Akt phosphorylation.

Although Se@SiO_2_ NPs have been proven to inhibit oxidative stress, which in turn could promote scar-free wound healing, the effect of reduced ROS on other cellular components in the wound microenvironment is unknown. We found that Se@SiO_2_ NPs could accelerate the re-epithelialization of wounds histologically, but we did not further explore the role of epithelial cells in this process. In future studies, we would investigate the nanomaterial–epithelial or matrix–epithelial interaction in the process of wound healing. Additionally, we ignored the effect of CS, which has an anti-inflammatory effect. As mentioned previously, inflammatory factors also changed with Se@SiO_2_ NP treatment, but we did not consider this change. Other potential effects of Se@SiO_2_ NPs that are conducive to wound repair, such as antibacterial effects, should be further explored. Last but not least, the PI3K/Akt pathway was researched in this study, while other signaling pathways involved in the Se@SiO2 NPs mediated wound repair promotion are still awaited to be explored.

## 5 Conclusion

In the current study, our team demonstrated that the administration of Se@SiO_2_ NPs could accelerate the re-epithelization of wound tissues, thus promoting dermal wound healing in rats. Additionally, the application of these NPs to wounds was effective in decreasing scar formation during recovery. We confirmed that Se@SiO_2_ NPs suppressed oxidative stress levels *in vivo* and *in vitro* with biosafety. Se@SiO_2_ NPs improved wound repair and optimized fibrotic tissue structure by reducing the high levels of ROS associated with inflammatory responses. Moreover, we also proved that Se@SiO_2_ NPs prevented apoptosis and blocked the differentiation of fibroblasts through PI3K/Akt pathway suppression. In summary, the findings of this study illustrate a new strategy in which treatment with Se@SiO_2_ NPs could shorten the healing process and enhance scarless dermal wound healing.

## Data Availability

The original contributions presented in the study are included in the article/Supplementary Material, further inquiries can be directed to the corresponding author/s.
