# Supplementary figures and images for: Porous Se@SiO2 Nanoparticles Enhance Wound Healing by ROS-PI3K/Akt Pathway in Dermal Fibroblasts and Reduce Scar Formation
Source: Front Bioeng Biotechnol. 2022 Mar 21;10:852482. doi: 10.3389/fbioe.2022.852482 (PMC8978548; doi:10.3389/fbioe.2022.852482)

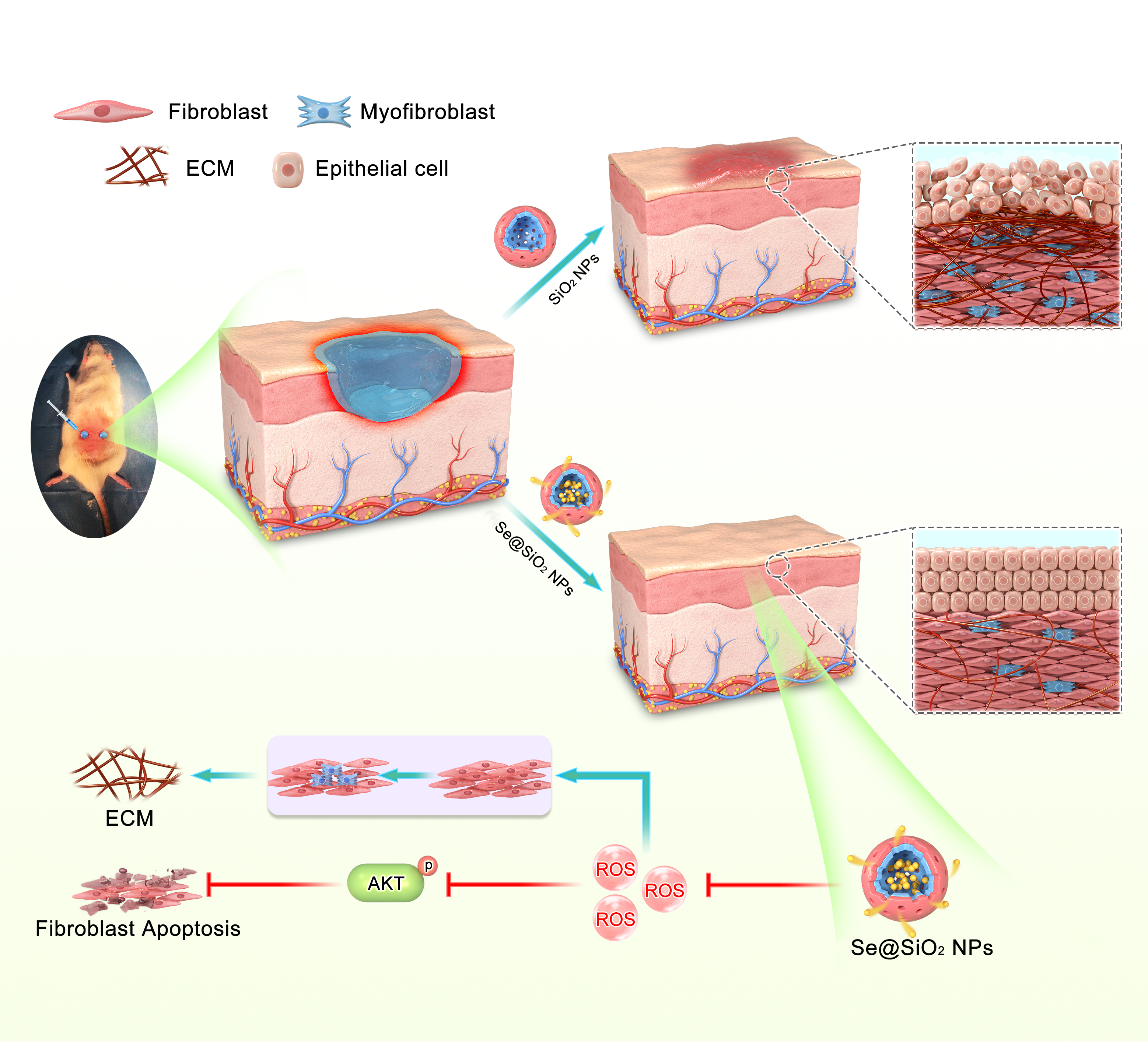

Supplement: Supplementary file 1 [file Image1.PNG]
