# Supplementary material for: Porous Se@SiO2 Nanoparticles Enhance Wound Healing by ROS-PI3K/Akt Pathway in Dermal Fibroblasts and Reduce Scar Formation
Source: Front Bioeng Biotechnol. 2022 Mar 21;10:852482. doi: 10.3389/fbioe.2022.852482 (PMC8978548; doi:10.3389/fbioe.2022.852482)

Table S1. Serum selenium content in Control and Experimental group was detected by ICP-AES weekly.


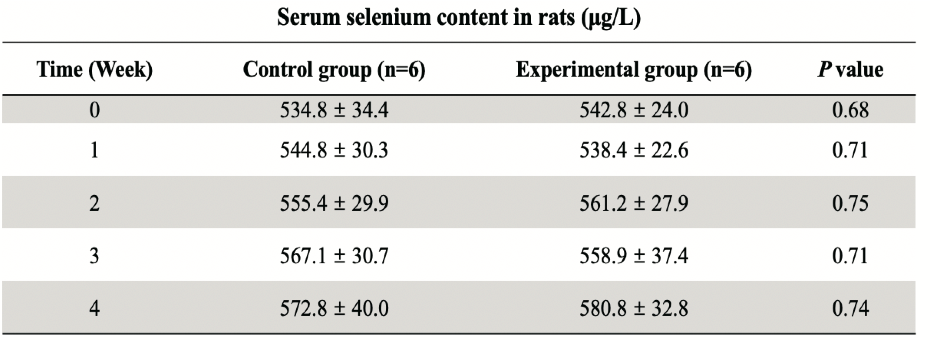


Table S2. Liver and kidney function of rats in Control and Experimental group at Day 28 was showed. Abbreviations: ALB, albumin; ALP, alkaline phosphatase; ALT, alanine transaminase; AST, aspartate transaminase; BUN, blood urea nitrogen; Cre, creatinine; γ-GT, γ-glutamyl transpeptidase; UA, uric acid; TBIL, total bilirubin.


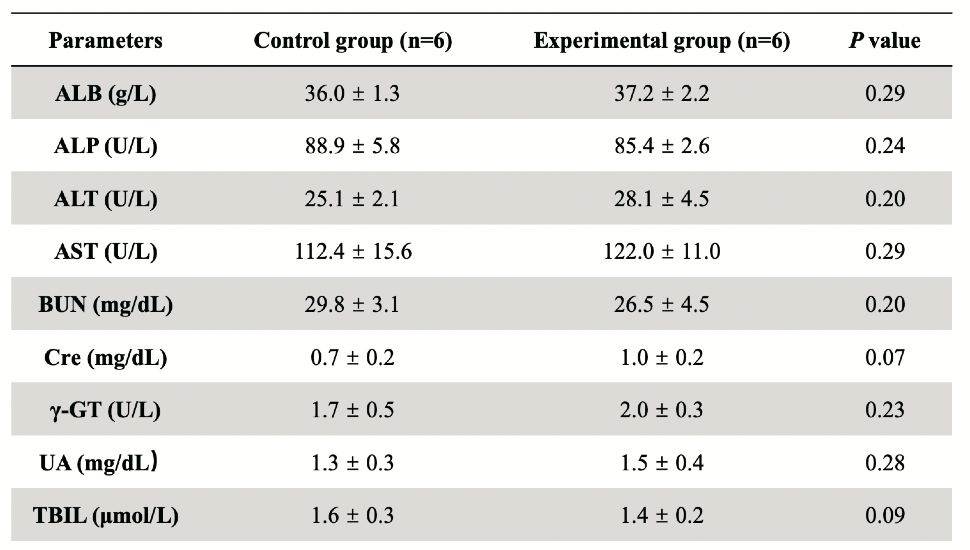

Supplement: Supplementary file 2 [file DataSheet1.docx]
